# A case series of early and late cranioplasty—comparison of surgical outcomes

**DOI:** 10.1007/s00701-019-03820-9

**Published:** 2019-02-04

**Authors:** Anna Bjornson, Tamara Tajsic, Angelos G. Kolias, Adam Wells, Mohammad J. Naushahi, Fahim Anwar, Adel Helmy, Ivan Timofeev, Peter J. Hutchinson

**Affiliations:** 1 Division of Neurosurgery, Department of Clinical Neurosciences, Addenbrooke’s Hospital & University of Cambridge, Cambridge, UK; 2 Department of Rehabilitation Medicine, Cambridge University Hospitals NHS Foundation Trust, Cambridge, CB20QQ UK

**Keywords:** Neurosurgery, Complications, Cranial reconstruction, Craniectomy

## Abstract

**Background:**

Cranioplasty is an increasingly common procedure performed in neurosurgical centres following a decompressive craniectomy (DC), however, timing of the procedure varies greatly.

**Objectives:**

The aim of this study is to compare the surgical outcomes of an early compared to a late cranioplasty procedure.

**Methods:**

Ninety adult patients who underwent a prosthetic cranioplasty between 2014 and 2017 were studied retrospectively. Timing of operation, perioperative complications and length of stay were assessed. Early and late cranioplasties were defined as less or more than 3 months since craniectomy respectively.

**Results:**

Of the 90 patients, 73% received a late cranioplasty and 27% received an early cranioplasty. The median interval between craniectomy and cranioplasty was 13 months [range 3–84] in late group versus 54 days [range 33–90] in early group. Twenty-two patients in the early group (91%) received a cranioplasty during the original admission while undergoing rehabilitation. Complications were seen in 25 patients (28%). These included wound or cranioplasty infection, hydrocephalus, symptomatic pneumocephalus, post-operative haematoma and cosmetic issues. The complication rate was 21% in the early group and 30% in the late group (*P* value 0.46). There was no significant difference in the rate of infection or hydrocephalus between the two groups. Length of stay was not significantly increased in patients who received an early cranioplasty during their initial admission (median length of stay 77 days versus 63 days, *P* value 0.28).

**Conclusion:**

We have demonstrated the potential for early cranioplasty to be a safe and viable option, when compared to delayed cranioplasty.

## Introduction

Cranioplasty is a common neurosurgical procedure performed to reconstruct a skull defect. It is most commonly performed following a decompressive craniectomy (DC), a procedure performed for raised intracranial pressure (ICP), which can occur following traumatic brain injury (TBI), cerebral infarction, subarachnoid haemorrhage, intracerebral haemorrhage, encephalitis and venous sinus thrombosis [14]. Other indications for cranioplasty include following the removal of bone-invading tumours or an infected bone-flap [14].

An increasing number of cranioplasty procedures have been performed in recent years due to the rising popularity of DC to manage raised intracranial pressure following traumatic brain injury and malignant ischaemic stroke [14]. One recent large randomised controlled trial has demonstrated reduced mortality in TBI patients with refractory raised ICP following DC [11]. Several large randomised controlled trials have demonstrated reduced mortality and improved outcomes following DC in malignant ischaemic stroke [1, 9, 13, 24, 29], and decompression is now part of the national guidelines for stroke management [1]. All of this has resulted in a growing cohort of patients who subsequently require a reconstructive cranioplasty.

The aim of a cranioplasty is to restore cosmetic appearances, protect the underlying brain from further injury and facilitate neurological recovery and rehabilitation [21]. A number of mechanisms have been proposed for reversible neurological disability following craniectomy—this includes cerebrospinal fluid flow disruption, venous sinus congestion, abnormal atmospheric pressures which can lead to ‘syndrome of the trephined’ and alterations in cellular metabolism [4, 15]. There is evidence for reduction in cerebral blood flow following DC, demonstrated by perfusion CT and transcranial Doppler ultrasound, which is attributed to the effect of atmospheric pressure on the decompressed area of brain [8]. Consequently, cranioplasty has been shown to resolve the cerebral blood flow and lead to improvement in neurological function [8, 10, 18, 23, 25].

There is a considerable variation in practice in terms of timing of cranioplasty following DC [7, 21, 25]. Traditionally, a cranioplasty would be delayed to allow for cerebral oedema to resolve and for the patient's neurological status to improve, and to reduce the chance of wound infection and delayed hydrocephalus [14]. There is evidence from a number of retrospective studies suggesting that an early cranioplasty procedure may lead to similar or reduced complication rates [2, 3, 6, 12, 20, 22, 28], with an improvement in overall neurological outcome [18, 20, 23, 28]. This has been supported by two systematic reviews [18, 27]. On the contrary, there is also evidence that infection and the development of hydrocephalus are more prevalent when cranioplasty is performed later [5, 17, 19, 26]. Notably, these studies varied in their definition of ‘early’ cranioplasty ranging from 6 weeks to 3 months, however, both of the systematic reviews used 3 months as the divider between early and late. Another study looked at preoperative CT findings and found that the brain sunken ratio was a stronger predictor of postoperative complications [16], suggesting that cranioplasty timing should take into account individual patient factors rather than having a set time for all patients. Overall, there does not seem to be a clear consensus on whether cranioplasty can safely be performed early and whether it may confer additional benefits in terms of neurological recovery.

In our neurosurgical unit, a cranioplasty is traditionally performed at 6–12 months to allow the wound to heal and for the patient to recover from the acute insult. However, the growing evidence in support of an earlier cranioplasty has led to some surgeons electing to perform the cranioplasties at an earlier date. This allows a direct comparison between the groups to assess outcomes and complications.

We present a retrospective study of patients undergoing cranioplasty following DC. We compare the effect of timing of cranioplasty on complications and also demonstrate how an early cranioplasty procedure can be safely performed during the initial hospital admission, with reduction in the overall length of hospital stay.

## Methods

A retrospective review was performed on all patients who underwent a reconstructive cranioplasty at a single regional neurosurgical unit over a 3-year period between October 2014 and November 2017. Data was collected from the hospital’s electronic database. Individual patient consent and IRB/ethics approval was not required as the study was undertaken as an audit with local institutional approval.

Information analysed included patient age and sex, indication for craniectomy, cranioplasty material and construction method, interval between craniectomy and cranioplasty, length of hospital stay, perioperative complications and time to last follow-up. Patients who underwent a cranioplasty following removal of an infected bone flap after a craniotomy were excluded from the study.

Two groups were identified—those who had received an ‘early’ cranioplasty, defined as less than 3 months from craniectomy, and those who received a ‘late’ cranioplasty, defined as more than 3 months from craniectomy. Perioperative complications were compared across the two groups and statistical analysis was performed using a Fisher’s exact test. Length of hospital stay was calculated for each admission and compared between the groups.

## Results

### Patient and surgical factors

Ninety patients were included in the study. Sixty-three (70%) were male and 27 (30%) were female. Median age was 52 years, range 16–70 years. (Table 1).

**Table 1:** Demographics and surgical factors

Sex male/female	63 M: 27F
Median age (years)	52 (range 16-70)
Mean time to last follow-up (months)	8 (range 0–48)
Indication for craniectomy	Number of patients (%)
TBI with ASDH	34 (38%)
TBI	24 (27%)
Ischaemic stroke	27 (30%)
Intracerebral haemorrhage	3 (3%)
Cerebral abscess	1 (1%)
Subarachnoid haemorrhage	1 (1%)
Prosthetic material	Number of patients (%)
Titanium	89 (99%)
PEEK	1 (1%)

This table shows the demographic details collected on the patients, the different diagnoses requiring craniectomy and the prosthetic material used for cranioplasty

Indications for craniectomy included acute subdural haematoma (33 patients), secondary craniectomy for traumatic brain injury with raised intracranial pressure (22 patients), ischaemic stroke (26 patients), intracerebral haemorrhage (3 patients), cerebral abscess (1 patient) and aneurysmal subarachnoid haemorrhage (1 patient). All patients received a prosthetic cranioplasty—89 patients received a custom-made titanium cranioplasty and 1 patient received a PEEK plate (Table 1). Use of prosthetic cranioplasty material is standard practice in our department; therefore, no patients received an autologous cranioplasty.

The average time to last follow-up was 7.7 months (range 0–48). Nine patients did not receive follow-up. This included 3 patients who did not attend their outpatient appointments and 6 patients who died prior to follow-up (Table 1)

### Timing of cranioplasty

Of 90 patients, 66 patients (73%) received a late cranioplasty and 24 (27%) received an early cranioplasty.

The median interval between craniectomy and cranioplasty was 13 months [range 3–84] in the late group vs. 54 days [range 33–90] in the early group. No patients in the early group received a cranioplasty procedure before 1 month. Seven patients had a very long interval (longer than 24 months) between craniectomy and cranioplasty due to their initial injury occurring at another centre and the patients then being lost to follow-up before referral to our centre.

All patients in the late group were re-admitted electively for their cranioplasty. In the early group, 22 patients (91%) received a cranioplasty during their primary hospital admission, while undergoing rehabilitation, and 2 patients were re-admitted for the cranioplasty (Table 2).

**Table 2:** Timing of cranioplasty

	Number of patients (%)	Median time from craniectomy to cranioplasty
Early group	24 (27%)	54 days [range 33–90]
Late group	66 (73%)	13 months [range 3–84]

This table shows the median time interval between craniectomy and cranioplasty for the two groups. The early group is defined as time interval less than 3 months, and the late group is defined as greater than 3 months

Timing of the cranioplasty was determined by surgeon preference. At this time, most surgeons were continuing with traditional practice of waiting at least 6 months before performing cranioplasty. However, other surgeons would consider performing a cranioplasty earlier than 3 months based on the degree of recovery from their initial injury and operation, whether the patient had remained as an inpatient within the regional neuroscience centre to receive rehabilitation in the acute trauma rehabilitation ward, and also whether the cranioplasty plate had been manufactured and was ready for implantation. This has led to a selection bias, with the majority of early cranioplasty patients having trauma as their underlying pathology as the stroke patients were often transferred back to their local stroke unit a few days after the craniectomy.

Any significant preoperative disorders were recorded. Two patients were noted to have developed ‘syndrome of the trephined’ and ‘abnormal CSF dynamics’. One received a cranioplasty at 8 months and was included in the late cohort, the other received a cranioplasty at 46 days and was included in the early cohort. There was no significant difference in preoperative clinical condition in the two groups.

### Complications

Overall, complications were seen in 25 patients (28%). This included wound or cranioplasty infection (11 patients), hydrocephalus (7 patients), symptomatic pneumocephalus (3 patients), acute post-operative haematoma (2 patients), incompatible cranioplasty plate due to size (1 patient) and cosmetic issues (1 patient).

The complication rate was 5/24 in the early group (21%) and 20/66 in the late cranioplasty group (30%). There was no significant difference between the two groups (*P* = 0.46, Fisher’s exact test). Infection was defined as either superficial (requiring antibiotics only) or deep infection (requiring plate removal). Overall rate of infection (including superficial and deep infection) occurred in 8% of the early group and 13% of the late group (no significant difference, *p* > 0.99, Fisher’s exact test). Infection which required plate removal occurred in 8% of the early and 11% of the late group (no significant difference, *p* > 0.99, Fisher’s exact test). Hydrocephalus requiring ventricular shunting occurred in 8% on the early group and 8% of the late group (no significant difference, *p* > 0.99, Fisher’s exact test) (Table 3)

**Table 3:** Complications—early and late group

	Early	Late	Total
Number of patients	24	66	90
Number of patients with complications	5 (21%)	20 (30%)	25
Infection (total)	2 (8%)	9 (13%)	11
Infection requiring plate removal	2 (8%)	7 (11%)	9
Hydrocephalus—requiring ventricular shunt insertion	2 (8%)	5 (8%)	7
Pneumocephalus	1 (4%)	2 (3%)	3
Cosmetic issues	0	1 (2%)	1
Acute post-operative haematoma	0	2 (3%)	2
Inappropriate plate size	0	1 (2%)	1

This table shows postoperative complications recorded, divided into type of complication and the number of each complication in the early and late groups

### Length of stay

Patients who were admitted electively for a cranioplasty in a separate admission from their craniectomy had a median length of stay of 2 days (range 1–161 days). All patients attended preoperative assessment clinic prior to their admission.

Patients who underwent an early cranioplasty as part of the same admission as their craniectomy, had a median length of stay of 77 days (range 37–112 days). For comparison, we calculated the length of stay for decompressive craniectomy patients who underwent inpatient rehabilitation without an early cranioplasty, and this was found to be 63 days (range 26–178) (Table 4). This difference in length of stay is not statistically significant (*P* = 0.28), suggesting that performing an early cranioplasty during the same admission does not necessarily prolong the length of stay for patients undergoing inpatient rehabilitation. This only included data for TBI patients, as patients with ischaemic stroke were transferred to their local district general hospital for further rehabilitation.

**Table 4:** Length of stay

	Median length of stay
Early group (cranioplasty same admission)	77 days [range 37–112]
Late group (no cranioplasty during admission)	63 days [range 26–178]
Elective admission for cranioplasty	2 days [range 1–161 days]

This table shows the median length of stay for patients in the early and late groups. There was no significant difference in length of stay for patients who received a cranioplasty during their initial admission compared to those who did not. Patients who returned for an elective cranioplasty had an average length of stay of 2 days

## Discussion

This study provides further evidence that early cranioplasty performed at less than 3 months post-craniectomy does not lead to an increased risk of complications. We chose 3 months as the cut-off between early and late, as this is consistent with the recent major systematic reviews on this topic [17, 18, 26, 27].

In our study, there was no significant difference in complication rate between the early and late cranioplasty groups (21 and 30% respectively, *P* = 0.46). There was similarly no significant difference in the rate of specific complications between the two groups. We have deliberately collected thorough and comprehensive data on complications in order to accurately reflect the morbidity associated with cranioplasty. The complication rate may appear high due to the inclusion of superficial wound infection, symptomatic pneumocephalus and cosmetic issues—none of these complications required reoperation.

Several retrospective studies which also compared early and late cranioplasty have reported similar findings, however one retrospective study found an increased risk of infection in early cranioplasty [5], and two systematic reviews found an increased risk of hydrocephalus in early cranioplasty compared to late [17, 26]. These results are contradicted by a further review which did not find any increase in complications with early cranioplasty [27]. All of the reviews were limited by the retrospective nature of all papers included and differing definitions of the timing of the early and late cranioplasties. This suggests that further high-quality evidence is needed to determine the risk of complications in early and late cranioplasty. Our study did not show a significant increase in the risk of hydrocephalus with early cranioplasty. Hydrocephalus requiring ventricular shunting occurred in 8% on the early group and 8% of the late group in our study.

An additional benefit to an early cranioplasty procedure is the neurological improvement that is associated with cranioplasty. Several studies and a systematic review have demonstrated an improvement in neurological function following cranioplasty [8, 10, 15, 18, 23] and these improvements may be enhanced by an early cranioplasty. This is demonstrated in a systematic review which looked at eight separate studies and found early cranioplasty is associated with greater neurological recovery across all outcome measures [15]. Proposed mechanisms for improvement in neurological function following cranioplasty include the restoration of normal CSF dynamics and normal cerebral blood flow following reconstruction of the skull defect [4, 8, 10, 15, 18, 23]. This raises the tantalising prospect that early cranioplasty can augment the rehabilitation potential of patients, reducing the time required for recovery and improving outcomes. Our study did not assess neurological outcomes comparing the early and late cranioplasty patients, however, this would be a useful area for further research.

Our study has demonstrated that when cranioplasty is performed early, it can occur during the initial admission while the patient is undergoing inpatient rehabilitation. This negates the need for a further admission, which would include a pre-admission assessment, further blood tests and a median hospital stay of 2 days. We have demonstrated that if the cranioplasty is performed early and during the same admission, it will not prolong the inpatient length of stay. We acknowledge that the ability to perform a cranioplasty during a patient’s initial admission while undergoing inpatient rehabilitation depends on how the individual healthcare system is set up; however, it is not unusual for traumatic brain injury patients to have a prolonged inpatient stay due to their rehabilitation needs or while they are awaiting a rehabilitation placement. This study has shown that in cases where the inpatient stay is prolonged due to rehabilitation needs, an early cranioplasty can prevent the need for future readmission without prolonging the length of stay for the initial admission.

The limitations of this study include its retrospective nature and relatively small sample size. All series of this sort are likely to suffer from selection bias given the complexity of rehabilitation following severe TBI. We would envisage that the only mechanism for definitively addressing this question is a randomised controlled trial, notwithstanding the additional resource required to complete this.

## Conclusion

We propose that early cranioplasty performed less than 3 months from decompressive cranioplasty can be a safe alternative to delayed cranioplasty and can be performed in the initial admission while the patient is undergoing inpatient rehabilitation. The patients may benefit from enhancement of their neurological rehabilitation following the cranioplasty. Other benefits would include completing their treatment prior to discharge, reducing the risk of further injury to the cranial defect after discharge and improving cosmesis, and preventing the need for a further elective admission with preoperative assessments and a median length of stay of 2 days.
